# Interventional Treatment for Cholangiocarcinoma

**DOI:** 10.3389/fonc.2021.671327

**Published:** 2021-06-29

**Authors:** Hang Li, Li Chen, Guang-Yu Zhu, Xijuan Yao, Rui Dong, Jin-He Guo

**Affiliations:** Center of Interventional Radiology and Vascular Surgery, Department of Radiology, Zhongda Hospital, Medical School, Southeast University, Nanjing, China

**Keywords:** cholangiocarcinoma, interventional, intra-arterial therapies, ablation, brachytherapy

## Abstract

Cholangiocarcinoma (CCA) is the second most common type of primary liver malignancy. The latest classification includes intrahepatic cholangiocarcinoma and extrahepatic cholangiocarcinoma, with the latter one further categorized into perihilar and distal cholangiocarcinoma. Although surgical resection is the preferred treatment for CCA, less than half of the patients are actually eligible for radical surgical resection. Interventional treatment, such as intra-arterial therapies, ablation, and brachytherapy (iodine-125 seed implantation), has become an acceptable palliative treatment for patients with unresectable CCA. For these patients, interventional treatment is helpful for locoregional control, symptom relief, and improving quality of life. Herein, in a timely and topical manner, we will review these advances and highlight future directions of research in this article.

## Introduction

Cholangiocarcinoma (CCA) originates from the epithelial cells of the biliary tree. As the second most common type of primary liver malignancy globally, it accounts for approximately 3% of gastrointestinal tumors and 10–25% of hepatobiliary malignancies (1). The global mortality and incidence of CAA are currently trending upwards (2). Various guidelines have been modified to guide treatment strategies as well as to predict prognosis for patients with CCA (3). The latest classification is made based on the anatomic location and includes intrahepatic cholangiocarcinoma (iCCA) and extrahepatic cholangiocarcinoma (eCCA), with the latter further categorized into perihilar (pCCA) and distal cholangiocarcinoma (dCCA) (2, 4, 5). CCA is a group of heterogeneous diseases with different anatomy, epidemiology, etiology, pathogenesis, clinical presentations, and treatment modalities (4). The incidence of CCA varies by region, with more than 80% cases found in Asia and South America (6). Due to its aggressiveness, CCA is characterized by a high mortality rate and poor prognosis (1). Most patients with CCA present with several segments or vascular invasion and even local/distant metastasis. The 5-year survival of patients with CCA ranges from 20 to 35% (4, 7, 8). Currently, gemcitabine plus cisplatin (GC) is a standard chemotherapy regimen for unresectable CCA. Nevertheless, the prognosis is poor, with the median overall survival (OS) less than 1 year (9).

Immunotherapy has radically changed therapeutic algorithms of several hematologic and solid tumors (10). But in CCA treatments, immune checkpoint inhibitors still showed conflicting results (11–16). In several trials testing immunotherapy in anatomically and molecularly unselected CCA patients, immune checkpoint inhibition monotherapy has been disappointing (17, 18). Genomic studies have paved the way towards the identification of a wide number of possible targets, the most promising of which are fibroblast growth factor receptor (FGFR) aberrations and isocitrate dehydrogenase (IDH) mutations (19–22).

The use of liver transplantation to treat iCCA remains controversial owing to high early tumor recurrence rates and poor survival (23). It is contraindicated by the International Liver Cancer Association owing to a paucity of strong published evidence (4). For pCCA, currently, the strongest evidence comes from a multicenter study published in 2018 based on a large number of patients with pCCA (n = 304). The median OS of patients in the liver transplantation group was longer than that in the resection group (32.5 *vs.* 27.4 months, p = 0.049) (24). Also, a randomized controlled trial comparing liver transplantation with surgical resection is currently ongoing in France (NCT02232932).

Interventional treatment, such as intra-arterial therapies (IAS), ablation, and brachytherapy *via* iodine-125 (^125^I) seed implantation, has developed over the last decades and become an acceptable palliative treatment for patients with unresectable CCA. Active local treatment can help with locoregional control and symptom relief and therefore improves patients’ quality of life (25, 26). This article aims to explore the role of interventional therapy in the treatment for patients with unresectable CCA.

## Interventional Treatment for CCA

### Intra-Arterial Therapies

Anatomically, the normal liver receives 70–80% of its blood supply from the portal vein and 20–30% from the hepatic artery. Arterially directed therapies, including conventional transarterial chemoembolization (cTACE), drug-eluting bead transarterial chemoembolization (DEB-TACE), and Yttrium-90 radioembolization (Y90-RE), are the most widely applied treatments for unresectable hepatocellular carcinoma (HCC) (27–30). Given that CCA is less vascularized than HCC and contains more fibrous connective tissue, the effects of chemotherapy and drug-eluting beads on tumors may be reduced in theory (31–33). However, the extant literature still suggests that palliative treatment for unresectable CCA is safe and effective for offering a potential survival benefit (34–37).

#### Hepatic Arterial Infusion Chemotherapy (HAIC)

Liver-directed therapy *via* a HAIC pump enables the delivery of high-dose chemotherapy directly into the liver (38). The liver’s dual blood supply preferentially delivers high doses of chemotherapeutic agents to the hepatic artery, which supplies nearly all the tumor’s blood flow, while blood delivered by the portal vein maintains the health of the non-neoplastic liver parenchyma (39, 40). Since the liver clears chemotherapy *via* first-pass metabolism, this approach diminishes systemic toxic effects (38). Given that advanced disease within the liver accounts for most unresectable cases, continuous HAIC is particularly well suited to cancers in the liver. Using floxuridine, a precursor of fluorouracil and the most active agent, in HAIC is helpful for achieving much higher tumor drug levels compared with systemic administration (41, 42).

##### iCCA

In 2011, Inaba et al. conducted a phase I/II study to ascertain the recommended dose of hepatic arterial infusion using gemcitabine (GEM) for iCCA (JIVROSG) (43). Dose-limiting toxicity and recommended dose of hepatic arterial infusion of GEM were determined as the primary endpoint. For a sample size of 13 patients who failed to reach the primary endpoint, none of them achieved complete response (CR), while one patient achieved partial response (PR), eight achieved stable disease (SD), and four achieved progressive disease (PD). The study could not prove improvement in test results using HAIC, but it demonstrated an acceptable disease control rate (DCR) Nevertheless, the study had several limitations such as the small sample size and monotherapy of gemcitabine. Ghiringhelli et al. followed with a study to evaluate the efficacy and tolerability of HAIC with gemcitabine plus oxaliplatin on unresectable iCCA, resulting in an overall response rate (ORR) of 66% and tumor control in 91% of the patients (44). The median progression-free survival (PFS) and OS were 9.2 and 20 months, respectively. In 2014, Kasai et al. reported a study that involved 20 patients with advanced iCCA for evaluating HAIC using 5-fluorouracil (5-FU) combined with subcutaneous administration of pegylated interferon α-2b (45). The 1- and 2-year survival rates were 53.7 and 14.3%, respectively. The median OS was 14.6 months. Konstantinidis and colleagues compared the outcomes of patients with unresectable iCCA treated with HAIC plus systemic chemotherapy (SYS) with those treated with SYS alone (46). Disease was confined to the liver in 104 patients who underwent combination therapy of HAIC plus SYS (n = 78) or SYS alone (n = 26). The ORR in the combined group was better than that in the group receiving SYS alone, although this did not reach statistical significance (59 *vs* 39%, *P* = 0.11). Median OS for the combined group was longer than that of the patients who received SYS alone (30.8 *vs* 18.4 months, *P* < 0.001). Recently, Cercek et al. reported the results of HAIC floxuridine plus systemic administration of gemcitabine and oxaliplatin in patients with unresectable iCCA treated at two medical centers (38). In this single-arm clinical trial including 38 patients, the median PFS was 11.8 months with a 6-month PFS rate of 84.1%. The median OS was 25.0 months, and the 1-year OS rate reached 89.5%. These studies suggest that HAIC appears to be highly active and tolerable in patients with unresectable iCCA. It offers the best outcomes in terms of tumor response and survival. Further evaluation is warranted to determine whether HAIC should be incorporated into first-line treatment for patients with unresectable iCCA.

##### eCCA

Due to its difficulty in visualizing tumor vascularity and tumor stain on hepatic arterial angiographic images, pCCA has been paid little attention to. To evaluate the efficacy and safety of HAIC of oxaliplatin and 5-FU for pCCA, Wang et al. conducted a prospective phase II study (47). The ORR reached 67.6%, which was much higher than expected. DCR of 89.2% was also higher than that from previously reported systemic chemotherapy. The median PFS, local PFS, and OS were 12.2, 25.0, and 20.5 months, respectively. However, very few reports have been published, and more trials are needed to further investigate these results.

#### Conventional Transcatheter Arterial Chemoembolization

##### iCCA

Typically, the majority of implementation researches on iCCA were retrospective due to the rarity of this disorder. Among the limited prospective studies, Kiefer et al. reported a two-center study (48). Sixty-two patients with unresectable iCCA were treated with cTACE using cisplatin, doxorubicin, and mitomycin mixed with polyvinyl alcohol particles. Imaging assessments were performed every 3 months. The results showed that median OS from first time of diagnosis was 20 months. The 1-, 2-, and 3-year survival rates of patients were 75, 39, and 17%, respectively. For patients treated with TACE plus systemic chemotherapy, median OS was 28 months, and DCR was 76%.

In another study conducted by Li et al., the established prognostic nomogram (iCCA nomogram) was used to perform survival risk stratification (8). A total of 553 patients who underwent hepatectomy for iCCA between January 2008 and February 2011 were included (49). Of these patients, 122 were treated with adjuvant TACE. In the whole cohort, the 5-year recurrence and OS rates between the TACE and non-TACE groups were significantly different (5-year recurrence: 72.9 *vs* 78.1%; OS: 38.4 *vs* 29.7%). According to survival risk stratification generated by the iCCA nomogram, only those in the lowest tertile (nomogram scores ≥77) benefited from adjuvant TACE (TACE *vs* non-TACE groups: 90.4 *vs* 95.9% for 5-year recurrence, 21.3 *vs* 6.2% for 5-year OS). The study suggests the potential of the established prognostic nomogram as a clinical predictor of whether patients with iCCA can benefit from adjuvant TACE following liver resection.

Another multicentric retrospective study between 2014 and 2017 analyzed 335 patients in 12 centers (50). The median OS of patients in the TACE plus surgery group was longer than that of patients in the surgery group (63 *vs* 18 months, *P* = 0.041). According to the TNM staging, stage II/III patients were able to benefit from TACE after surgery (*P* = 0.021). Subgroups stratified by risk factors showed that only adjuvant TACE following liver resection might be suitable for iCCA patients with two or fewer risk factors (*P* = 0.027).

Schernthan et al. presented dual-phase cone-beam computed tomography (DPCBCT) as a new technique for survival analyses in patients with unresectable iCCA (51). This retrospective study included 17 consecutive patients with iCCA who were treated with cTACE. All the patients underwent pre-procedural contrast-enhance magnetic resonance imaging of the liver. In addition, digital subtraction angiography (DSA) and DPCBCT [early-arterial phase (EAP) and delayed-arterial phase (DAP)] were performed immediately before cTACE. Of the 61 iCCA lesions, only 45.9% were depicted by DSA, whereas EAP- and DAP-CBCT yielded significantly higher detectability rates of 73.8 and 93.4%, respectively (*P* < 0.01). DPCBCT, especially DAP-CBCT, significantly improved the detectability of iCCA lesions during cTACE compared to DSA. Additionally, the evidence suggests that iCCA patients would benefit more from cTACE as age increases.

##### eCCA

The retrospective study conducted by Zheng et al. involved 72 patients with pCCA. The control group consisted of 35 patients who received simple biliary drainage tube placement and biliary stent implantation, while 37 patients were in the experimental group and treated with cTACE using cisplatin plus gemcitabine (52). Results showed that median OS in the study group was significantly higher than that in the control group (20 *vs* 10.5 months, *P* < 0.05), as was patency duration (15.6 *vs* 7 months, *P* < 0.05). The authors suggest that the combination of cTACE with radiotherapy can significantly prolong the survival of patients with pCCA.

#### Drug-Eluting Bead-Transcatheter Arterial Chemoembolization

In cTACE, the oily contrast medium Lipiodol is emulsified with chemotherapeutic drugs and administered into tumor-feeding branches of the hepatic artery (53). Lipiodol and chemotherapeutic drugs may be isolated after injection because emulsification between them is unstable, leading to poor treatment outcomes. DEB-TACE has been promoted to improve tumor drug concentrations and to enhance its antitumor efficacy without increasing the risk of systemic toxicity (54). To our knowledge, the use of DEB-TACE in treating eCCA has not yet been reported, so this review mainly focuses on the response of iCCA patients.

##### iCCA

Aliberti et al. reported a prospective research on DEB-TACE loaded with doxorubicin for the treatment of unresectable iCCA (55). The tumor response for the whole sample of 127 patients was PR in 19 (15%) patients, SD in 101 (80%), and PD in 7 (5%) patients 3 months after therapy. The DCR was 95 and 85% in the DEB and PEG groups, respectively. In a small retrospective study with 20 patients, Poggi et al. treated nine iCCA patients with chemotherapy plus DEB-TACE, and carried out a retrospective comparison with 11 patients treated with chemotherapy only (56). Approximately 44% of patients in the chemotherapy plus DEB-TACE group achieved PR, and 56% achieved SD. The median OS was 30 months for the chemotherapy plus DEB-TACE groups and 12.7 months for chemotherapy-only patients (*P* = 0.004), respectively. Propensity score‐matched analysis showed a significant survival benefit for patients who received chemotherapy plus DEB-TACE compared to those undergoing chemotherapy only. Recently, a prospective multicenter study including 37 patients with iCCA was conducted (57). For patients treated with DEB-TACE loaded with Adriamycin, about 8.1% of them achieved CR, while 59.5% achieved PR. This treatment provided an ORR of approximately 67.6% and a median OS of 376 days. Furthermore, the multivariate Cox proportional hazards model identified macrovascular invasion (portal or hepatic vein tumor thrombi) as a risk factor for poor prognosis in iCCA patients. In the case of mild adverse events such as abnormal liver function, DEB-TACE is well tolerated by patients. Eighty-eight unresectable iCCA patients who received DEB-TACE treatment with CalliSpheres (CSM) were retrospectively enrolled in a recent study (58). The tumor response of the whole sample of 88 patients was PR in 58 (65.9%) patients, SD in 19 (21.6%), and PD in 11 (12.5%) at 1 month after therapy. The median PFS and OS were 3.0 months and 9.0 months, respectively. DEB-TACE with CSM is safe, effective, and well tolerated for unresectable iCCA patients, providing a high DCR, low complication rate, and relative benefit in terms of survival.

#### Radioembolization

Y90-RE has been demonstrated to provide long-term survival benefits for patients with primary or secondary liver tumors (59). Microspheres impregnated with Y90 are delivered through the hepatic artery to tumors with preferential blood flow. After Y90-containing microspheres go through arterial catheters to the hepatic arteries that feed the tumor, it enables a high radiation dose in the tumor with minimal exposure to normal tissues.

The clinical importance and efficacy of Y90-RE in treating HCC patients are widely documented in the international literature (60, 61). However, very limited data are available on the use of Y90-RE in CCA patients.

In 2008, Ibrahim et al. was the first to research Y90-RE in treating patients with iCCA (62). Twenty-four patients with histologically proven iCCA were treated. Based on imaging follow-up of 22 patients, 6 (27%), 15 (68%), and 1 (5%) patient achieved PR, SD, and PD, respectively. The median OS for the entire cohort was 14.9 months. This research suggests that Y90-RE may be a therapeutic option for unresectable iCCA. Hoffman et al. reviewed 33 patients with unresectable iCCA who were treated with Y90-RE (63). Overall, 12 patients achieved PR, 17 achieved SD, and 5 achieved PD after 3 months. The median OS was 22 months. Median time to progression was 9.8 months. In regard to grading criteria after Y90-RE, Camacho et al. evaluated patient response to Y90-RE using the modified response evaluation criteria in solid tumors (mRECIST) and the European association for the study of the liver (EASL) guidelines (64). The prospective study included 21 patients with a median OS of 16.3 months. The efficacy of mRECIST and EASL was 56.2 and 50%, respectively, which were much higher than 6.2% generated by RECIST (*P* < 0.001). This research may propose an effective and feasible method for the assessment of iCCA patients treated with Y90-RE.

In general, regarding Y90-RE for treatment of iCCA, theexisting literature suggests that the treatment is effective and relatively safe. Additionally, it is well tolerated by patients with aggressive invasion. A substantial number of studies indicate that the ORR of Y90-RE in iCCA patients range from 24 to 82.3% (**Tables 1**, **2**) .

**Table 1:** Study design and characteristics within included studies for IAS.

Investigators	No. of patients		Study interval	Design	Diagnosis	Control group	Experimental group	Ref.
Inaba (2011)		13	2004–2005	Prospective	Unresectable iCCA	/	HAIC (GEM)	(43)
Ghiringhell (2013)		12	2008–2013	Retrospective	Unresectable iCCA	/	HAIC (GEM plus oxaliplatin)	(44)
Kasai (2014)		20	2008–2013	Prospective	Unresectable iCCA	/	HAIC (5-FU plus subcutaneous PEG-IFN α-2b)	(45)
Konstantinidis (2015)		104	2000–2012	Retrospective	Unresectable iCCA	Systemic chemotherapy alone	HAIC plus systemic chemotherapy	(46)
Cercek (2019)		38	2013–2019	Prospective	Unresectable iCCA	/	HAIC (floxuridine) plus systemic chemotherapy (gemcitabine and oxaliplatin)	(38)
Wang (2016)		37	2012–2015	Prospective	Unresectable pCCA	/	HAIC (oxaliplatin plus 5-FU)	(47)
Kiefer (2010)		62	N/A	Retrospective	Unresectable iCCA	/	cTACE (cisplatinum, doxorubicin, mitomycin-C, ethiodol, and polyvinyl alcohol particles)	(48)
Li (2015)		553	2008–2011	Retrospective	iCCA after hepatectomy	Non-cTACE (n = 431)	cTACE (n = 122)	(49)
Wang (2020)		335	2014–2017	Retrospective	iCCA after hepatectomy	Non-cTACE (n = 296)	cTACE (n = 39)	(50)
Zheng (2019)		72	2014–2018	Retrospective	Unresectable pCCA	Biliary drainage tube placement and biliary stent implantation (n = 35)	cTACE (gemcitabine and cisplatin) and extracorporeal radiotherapy after biliary drainage or biliary stent implantation (n = 37)	(52)
Aliberti (2017)		127	2000–2016	Prospective	Unresectable iCCA	N/A	DEB-TACE loaded with doxorubicin	(55)
Poggi (2009)		20	2005–2008	Retrospective	Unresectable iCCA	Chemotherapy	DEB-TACE loaded with oxaliplatin	(56)
Luo (2020)		37	2015–2016	Prospective	Unresectable iCCA	N/A	DEB-TACE loaded with Adriamycin	(57)
Zhou (2020)		88	2015–2018	Retrospective	Unresectable iCCA	N/A	DEB-TACE treatment with epirubicin	(58)
Ibrahim (2008)		24	2004–2008	Prospective	Unresectable iCCA	N/A	Y90-RE	(62)
Hoffmann (2011)		33	2007–2010	Retrospective	Unresectable iCCA	N/A	Y90-RE	(63)

iCCA, intrahepatic cholangiocarcinoma; pCCA, perihilar cholangiocarcinoma; HAIC, hepatic arterial infusion chemotherapy; cTACE, conventional transcatheter arterial chemoembolization; DEB-TACE, drug-eluting bead transcatheter arterial chemoembolization; Y90-RE, yttrium-90 radioembolization; GEM, gemcitabine; 5-FU, 5-fluorouracil; PEG-IFN, pegylated interferon.

**Table 2:** Results and adverse events within included studies for IAS.

Investigators	ORR (EG or EG *vs* CG)	DCR (EG or EG *vs* CG)	PFS (EG or EG *vs* CG)	Median OS (EG or EG *vs* CG)	Adverse events (grade III/IV)
Inaba (43)	7.7% (95% CI, 0.2–36.0%)	69% (95% CI, 38.6–90.9%)	N/A	389 d (95% CI, 158–620)	Neutropenia (n = 2), elevated GGT (n = 1), elevated AST (n = 1), elevated ALT (n = 1), elevated bilirubin plus PVTT (n = 1)
Ghiringhell (44)	66.6% (95% CI, 29–100%)	91% (95% CI 45–100%)	9.2 mo (95% CI, 5.1–29.4)	20.3 mo (95% CI, 13.2–49.7)	Neutropenia (n = 2), thrombocytopenia (n = 2), oxaliplatin allergy (n = 2)
Kasai (45)	60%	90%	8.0 mo (95% CI, 3.1–14.5)	14.6 mo (95% CI, 5.5–16.8)	Leukopenia (n = 2), thrombocytopenia (n = 2), anemia (n = 2)
Konstantinidis (46)	59 *vs* 39%, *P* = 0.11	N/A	N/A	30.8 *vs* 18.4 mo (*P* <.001)	None
Cercek (38)	58%	84%	11.8 mo (1-sided 90% CI, 11.1)	25.0 mo (95% CI, 20.6–not reached)	4 patients (11%) had grade 4 toxic effects: portal hypertension (n = 1), gastroduodenal artery aneurysm (n = 2), infection in the pump pocket (n = 1)
Wang (47)	67.60%	89.20%	12.2 mo (95% CI, 6.60–17.83)	20.5 mo (95% CI, 11.12–29.88)	Severe anemia (16.2%), leukopenia (10.8%), thrombocytopenia (13.5%), Grades 3 and 4 liver enzyme elevation (8.1%), severe abdominal pain (5.4%)
Kiefer (48)	11%	76%	8 mo	20 mo	Pulmonary edema and elevated cardiac enzymes (n = 1), pulmonary infarct (n = 1), severe postembolization syndrome (n = 1), hyperglycemia (n = 1), acute renal failure and dehydration (n = 1)
Li (49)	N/A	N/A	22.0 *vs* 14.3 mo	27.6 *vs* 20.4 mo	None
Wang (50)	N/A	N/A	50 *vs* 10 mo, *P* = 0.022	63.0 *vs* 18.0 mo, *P* = 0.041	None
Zheng (52)	N/A	N/A	10.5 mo	20.0 mo	Biliary hemorrhage and cholangitis
Aliberti (55)	15%	95%	N/A	14.53 mo (95% CI, 9.17–15.23)	Nausea/vomiting (24%), fever (7%), pain (7%)
Poggi (56)	44%	100%	8.4 *vs* 2.9 mo, *P* = 0.1	30 *vs* 12.7 mo, *P* = 0.004	Abdominal pain (24%; 28/67), cholangitis G3 (3%; 2/67), hypertensive crisis G3 (3%; 2/67)
Luo (57)	67.60%	91.90%	N/A	376 d (95% CI, 341–412 d)	None
Zhou (58)	65.90%	87.50%	3 (95% CI, 2.5–3.5)	9 (95% CI, 7.0–11.0)	None
Ibrahim (62)	27%	95%	N/A	14.9 mo	Albumin toxicities (17%)
					Bilirubin toxicity (4%)
Hoffmann (63)	36.40%	87.90%	9.8 mo	22 mo	No clinical relevant acute or delayed toxicities

ORR, overall response rate; DCR, disease control rate; PFS, progression-free survival; OS, overall survival; CG, control group; EG, experimental group; 95% CI, 95% confidence interval; GGT, gamma-glutamyl transpeptidase; AST, aspartate aminotransferase; ALT, alanine aminotransferase; PVTT, portal vein tumor thrombosis.

### Ablation

Ablation has been a treatment option for HCC for advantages of minimal invasion, simple, maneuverable, low risk, and high cost-effectiveness (65–68). It is cheaper and quicker to perform than resection and reduces hospital admission (69). In 2019, Yousaf et al. reported a meta-analysis on ablative therapies treating iCCA (70). A total of 10 studies were included, yielding an aggregate of 206 patients and 320 tumors. The median OS ranged from 8.7 to 52.4 months. Pooled 1-, 3-, and 5-year survival rates were 76, 33, and 16%, respectively. Ablative therapies display promising potential as treatment modalities for CCA.

#### Radiofrequency Ablation (RFA)

Radiofrequency ablation is the most studied energy-based ablative method. It utilizes high-frequency alternating electric current to cause cell death by heating tissue through rapid electron vibration that generates frictional heat (71). RFA has been applied to treat esophageal and primary or malignant liver tumors. The length of hospital stay, treatment cost, and risk of complications tend to be lower with RFA than with surgery (72, 73).

##### iCCA

Seven studies and 84 patients were included in a recent meta-analysis to assess the efficacy and safety of RFA in treating iCCA (74). The pooled 1-, 3-, and 5-year survival rate was 82, 47, and 24%, respectively. The authors conclude that RFA is a locoregional treatment option that prolongs survival rates in patients with iCCA who are ineligible for surgery.

The efficacy of RFA is closely associated with tumor size. Carrafiello et al. obtained complete necrosis of all four tumors smaller than 4 cm. However, they were unable to achieve the same result in the two patients with tumors larger than 5 cm even though pre-interventional TACE was performed to increase the area of ablation (75). As a result, residual tumors were observed in these two patients with larger tumors (5 and 5.8 cm in diameter). All patients could tolerate the procedure, and no major complication was identified. In Kim et al.’s study on primary iCCA treated with RFA, technical effectiveness was achieved in all 11 patients with tumors <5 cm but in none of the patients with larger lesions (76). Giorgi et al. performed RFA on 10 patients with unresectable iCCA and proved its effectiveness for lesions smaller than 3.4 cm (77). However, for those larger than 4 cm, RFA failed to provide survival benefit. Recently, Brandi et al. reported a retrospective study including 29 patients with 117 nodules. At a median follow up of 39.9 months, median OS was 27.5 months. The 1-, 2-, and 4- years of OS was 89, 45, and 11%, respectively. It indicates that tumor size ≥20 mm was associated with lower PFS, representing a potential useful threshold value (78). To be concluded, for smaller iCCA lesions, percutaneous RFA should have a higher chance of success.

RFA guided by endoscopic retrograde cholangiopancreatography (ERCP) in treating iCCA and other intraductal malignancies is gaining popularity (79). Several studies demonstrate that patients receiving RFA with stenting maintained stent patency at 30 days, deeming endobiliary RFA to be a safe treatment (80, 81).

##### eCCA

Wang et al. treated nine patients with unresectable Bismuth types III and IV hilar cholangiocarcinoma using percutaneous intraductal RFA combined with metal stent placement after percutaneous transhepatic cholangial drainage (82). Median stent patency from the time of the first RFA and median OS were 100 days and 5.3 months, respectively. For dCCA, Wu et al. investigated the clinical efficacy of intraductal RFA in patients with dCCA (83). The RFA group had a longer median stent patency than the control group (*P* = 0.001). Prolonged stent patency, better functional status, and improved quality of life, which are all important clinical endpoints, were observed in patients treated with intraductal RFA.

#### Microwave Ablation (MWA)

MWA describes a thermoablative, minimally invasive technique for treatment of tumors. Its efficiency in solid tumors has been widely reported over the years and is concerned with many theoretical advantages, such as shorter ablation time, higher ablation temperature, shorter operative time, and larger ablation zone (84–86). To the best of our knowledge, there is no relevant literature on the use of MWA in treating patients with eCCA.

##### iCCA

A prospective study in 2011 involved 15 unresectable iCCA patients (87). All patients received ultrasound-guided percutaneous MWA. The rates for ablation success, technique effectiveness, and local tumor progression were 91.7% (22/24), 87.5% (21/24), and 25% (6/24), respectively. Minor complications and side effects were experienced by most patients, which subsided with supportive treatment. A retrospective analysis conducted by Zhang et al. showed that in a cohort of 107 patients (88), the frequency of major complications was 2.8% and that of procedure-related deaths was 0%. The median PFS and median OS were 8.9 months and 28.0 months, respectively. The 1-, 3-, and 5-year OS rates were 93.5, 39.6, and 7.9%, respectively. To compare the effectiveness of the two therapeutic methods (MWA and surgery), Xu et al. reported a retrospective study with 121 patients included (89). Fifty-six patients were treated with MWA, while 65 underwent surgery. The OS and RFS after MWA were comparable to those after surgery (*P* = 0.405 and *P* = 0.589, respectively). The estimated 5-year OS rate was 23.7% after MWA and 21.8% after surgery; for RFS, the estimated 3-year RFS rate was 33.1% after MWA and 30.6% after surgery. Major complication rate in the surgery group was higher than that in the MWA group (*P* < 0.001) (13.8 *vs* 5.3%). The authors suggest that MWA produces oncologic outcomes comparable to those of surgery and could be a safe and effective treatment for recurrent iCCA after hepatectomy.

#### Irreversible Electroporation (IRE)

In 2005, IRE was first introduced as a non-thermal ablation technique for tumor ablation (90). The electrical pulses permeabilize the lipid bilayer of the cell membrane, thereby disrupting intracellular homeostasis and inducing apoptosis (91–93). In contrast to other thermal ablation techniques, IRE work through a high-voltage pulsed electric field without destructing the extracellular matrix (94). Various retrospective studies have demonstrated the safety of peribiliary tumor ablation with IRE (95, 96), and catheter-directed IRE in the swine bile duct was designed for biliary malignancies (97). IRE is a relatively new technology and therefore, very few studies have been conducted so far.

##### iCCA

The very few studies on iCCA include Belfiore et al.’s investigation which assessed the safety, feasibility, and efficacy of IRE in treating CCA (98). A total of 15 patients with unresectable CCA (eight with iCCA and seven with pCCA) were enrolled in this prospective study. The imaging follow-up showed local disease control with a decrease in the entire volume of the lesion and a further reduction in the densitometric values. The mean survival was 18 months (95% CI, 18.8–36.7 months).

##### eCCA

In 2018, Martin et al. conducted a study to investigate the safety and efficacy of IRE in treating obstructive jaundice in advanced pCCA (99). Twenty-six patients underwent IRE for pCCA after percutaneous transhepatic biliary drainage (PTBD) replacement. After treatment, the median time to PTBD removal was 122 days (ranging 0–305 days), and the median catheter-free time before requiring PTBD replacement was 305 days (ranging 92–458 days). Authors suggest that in patients with pCCA who present with obstructive jaundice, IRE can be used to increase catheter-free days and to optimize overall quality of life. To assess the safety and efficacy of IRE for unresectable pCCA, Hsiao et al. treated nine patients from two medical centers in Asia using IRE (100). The median OS was 26 months, and the median PFS was 18 months. These studies show that IRE ablation of unresectable CCA involving vital structures is a safe and feasible primary treatment for local tumor control and is effective in prolonging survival.

By far, multicenter large prospective studies are still lacking. However, a randomized controlled trial in a multicenter phase I/II safety and feasibility study is underway (101) (**Tables 3**, **4**).

**Table 3:** Study design and characteristics within included studies for ablation.

Investigators	No. of patients	No. of tumors	Tumor size	Study interval	Design	Diagnosis	CG	EG	Ref.
Carrafiello (2010)	6	7	1–5.8 cm	2004–2008	Prospective	Unresectable iCCA	/	RFA	(75)
Kim (2011)	13	17	0.8–8 cm	2000–2009	Retrospective	Unresectable iCCA	/	RFA	(76)
Giorgi (2011)	10	12	2.4–7.0 cm	2003–2010	Retrospective	Unresectable iCCA	/	RFA	(77)
Brandi (2020)	29	117	0.5–4.8cm	2014–2019	retrospective	unresectable iCCA	/	RFA	(78)
Yu (2011)	15	24	1.3–9.9 cm	2006–2010	Prospective	iCCA	/	MWA	(87)
Zhang (2017)	107	171	<5 cm	2009–2016	Retrospective	Recurrent iCCA	/	MWA	(88)
Xu (2019)	121	136	<5	2011–2017	Retrospective	Recurrent iCCA	SR (n = 65)	MWA (n = 56)	(89)
Belfiore (2020)	8	8	5.6–267.2 cm^3^	2015–2019	Prospective	Unresectable iCCA	/	IRE	(98)
	7	7	23.4–159.5 cm^3^	2015–2019	Prospective	Unresectable pCCA			

iCCA, intrahepatic cholangiocarcinoma; pCCA, perihilar cholangiocarcinoma; RFA, radiofrequency ablation; MWA, microwave ablation; SR, surgical resection; IRE, irreversible electroporation; CG, control group; EG, experimental group.

**Table 4:** Results and adverse events within included studies for ablation.

Investigators	Follow-up (mo) (range)	Technical success	Technical effectiveness	PFS (EG or EG *vs* CG)	Median OS (EG or EG *vs* CG)	Adverse events (grade III/IV)
Carrafiello (75)	Mean 17.5 (13–21)	/	66	/	/	None
Kim (76)	Median 19.5 (3.3–82.1)	88%	88%	32.2 mo	38.5 mo	Liver abscess (n = 1)
Giorgi (77)	Median 19.5 (9-64)	/	66	/	/	None
Yu (87)	Mean 12.8 (4–31)	91.70%	87.50%	/	10 mo	Liver abscess (13.3%), needle seeding (6.7%)
Zhang (88)	Median 20.1 (2.8–63.5)	100%	93.00%	8.9 mo (95% CI,6.5–11.3)	28 mo (95% CI, 23.7–32.2)	Pleural effusion (1.9%), liver abscess (0.9%)
Xu (89)	/	100 *vs* 100%	100 *vs* 100%	*P* = 0.589	31.3 *vs* 29.4, *P* = 0.405	MWA: hepatic failure (n = 1), liver abscesses (n = 2), ascites (n = 1)
						SR: hepatic failure (n = 2), ascites (n = 6), jaundice (n = 3)
						MWA *vs* SR: 5.3 *vs* 13.8% (*P* < 0.001)
Belfiore (98)	(6–48)	100%	100%	/	18 mo (95% CI, 18.8–36.7)	None

PFS, progression-free survival; OS, overall survival; MWA, microwave ablation; SR, surgical resection; CG, control group; EG, experimental group.

#### Cryoablation

Cryoablation produces intra- and extracellular ice crystals to cause dehydration and osmotic pressure changes of the cells. These changes induce the damage of cell membrane and organelle and leads to cell death eventually.

Currently, few studies have evaluated the effectiveness of cryoablation for the treatment of CCA. In a retrospective study, 299 hepatic tumors were included (102). The technical success rate was 94.6% (279/295). The technique efficacy rate was 89.5% (231/258) and was greater for tumors smaller than 4 cm (93.4%; 213/228) than for larger tumors (60.0%; 18/30) (*P* < 0.0001). However, only six patients in this study had CCA. Another study on cryoablation involved 39 hepatic tumors in total, but only three were iCCA. These studies suggest that cryoablation provides an option for hepatic tumors, but compelling evidence for CCA remains insufficient. Further studies focusing on cryoablation of CCA may help demonstrate its role in a select group of patients where resection or other ablative therapies are not possible (103).

### Brachytherapy (^125^I Seed Implantation/^125^I Seeds Combined With Biliary Stenting)

In 2006, Nag et al. reported a study of 64 patients with unresectable primary and metastatic liver tumors from 1989 to 2002 (104). Patients were treated with ^125^I brachytherapy of 160 Gy. The overall 1-, 3-, and 5-year actuarial intrahepatic local control rates were 44, 22, and 22%, respectively, with a median time to recurrence of 9 months (95% CI, 6–12 months). The 1-, 3-, and 5-year actuarial OS rates were 73, 23, and 5%, respectively (median, 20 months; 95% CI, 16–24). Back to that time, primary liver carcinoma widely included HCC and intrahepatic bile duct carcinoma. However, this study still shows potential benefit for iCCA patients.

When CCA causes malignant biliary obstruction, percutaneous transhepatic-cholangiodrainage and percutaneous biliary stent (PTBS) can mitigate the complications of further biliary obstruction, reduce serum bilirubin, and improve quality of life. As an effective method of palliation, PTBS leads to a shorter hospital stay and decreased risk of postoperative complications compared with surgery (105). However, stents can become occluded due to epithelial hyperplasia, tumor ingrowth or overgrowth, biofilm deposition, and sludge formation; thus, this process may need to be repeated several times. In contrast, ^125^I seeds combined with biliary stenting can relieve stent blockage significantly (106, 107). In 2018, Zhu et al. reported a randomized, open-label trial of 328 participants with unresectable malignant biliary obstruction at 20 centers in China (108). The participants were allocated into the ^125^I seed-loaded irradiation stent group (ISG) or the uncovered self-expandable metallic stent (USG). The first quartile stent patency time (when 25% of the patients experienced stent restenosis) was 212 days for ISG and 104 days for USG. ISG was linked with a significant decrease in the rate of stent restenosis (9 *vs* 15% at 90 days; 16 *vs* 27% at 180 days; 21 *vs* 33% at 360 days; *P* = .010). Longer median OS was achieved in ISG (202 days *vs* 104 days; *P* = .020). As for technical success rate and the incidence of complications, no significant difference was observed (93 *vs* 95%, *P* = .499; 8.5 *vs* 7.9%, *P* = .841). Compared with separate biliary stents, ^125^I seed-loaded biliary stenting can significantly prolong the survival time, improve the stent patency, and improve the prognoses of CCA patients (109–111).

#### eCCA

Cui et al. retrospectively analyzed 73 cases of pCCA patients who underwent PTBS combined with ^125^I seed intracavitary irradiation (112). The serum levels of total bilirubin, direct bilirubin, alanine aminotransferase, aspartate aminotransferase, and alkaline phosphatase were significantly reduced, while albumin was significantly increased at 1 and 3 months postoperatively. The median survival time of the cohort was 12 months, and the 1-year survival rate was 53.1%. Recently, Pang et al. retrospectively reviewed 184 patents to compare the efficacy of PTBS only and PTBS combined with ^125^I particle implantation when treating advanced eCCA (113). Among these patients, 71 received PTBS, and 113 received additional implantation of ^125^I particles. Jaundice and liver function were significantly improved in all patients, especially in the PTBS + ^125^I group. There was no significant difference in the risk of postoperative complications between the two groups. Meanwhile, the risk of biliary reobstruction was significantly reduced in the PTBS + ^125^I group (19.5 *vs* 35.2%, *P* = 0.017). Patients in the PTBS + ^125^I group had a significantly better OS for both pCCA and dCCA. The authors suggest that for patients with advanced eCCA, PTBS combined with ^125^I particle implantation is superior to PTBS monotherapy in improving liver function, inhibiting biliary reobstruction, and prolonging survival.

## Discussion

In summary, although large-scale randomized controlled trials are relatively sparse, the current literature suggests that palliative local interventional treatment is a safe, effective, and feasible method for unresectable or recurrent CCA after hepatectomy. Also, promising preclinical evidence shows that palliative treatment can significantly prolong survival time and improve quality of life with fewer side effects.

Among various interventional methods, intra-arterial embolotherapy has been widely accepted for its selective targeting precision, which augments drug exposure of tumors localized in the liver without increasing systemic toxicity.

Y90-RE has become increasingly acceptable, while in terms of palliative treatment in CCA, cTACE remains the most common intra-arterial therapy. With few toxic side effects, intra-arterial embolotherapy can be well tolerated.

In HCC, ablation has been considered a first-line therapy in most guidelines around the world. In regard to CCA, some guidelines also suggest that ablation may benefit patients with unresectable CCA. Current studies mostly concentrate on the size of the tumor to be treated. No guidelines have shown a benefit of ablation for management of patients with different sizes of CCA. However, ablation could be a safe and effective method when surgery is forbidden.

Several studies indicate that after implantation of radioactive ^125^I particles, the percentages of CD3^+^ T, CD4^+^ T, natural killer, and regulatory T cells significantly increased in the peripheral blood of tumor patients. In addition, the concentrations of immunoglobulin (IgM, IgG, and IgA) and complements (C3 and C4) also increased, indicating that ^125^I particles may stimulate not only cellular immunity but also humoral immunity (114, 115). Current evidence suggests that ^125^I brachytherapy is a promising treatment and deserves more attention to further investigation.

Many studies document the limitations of current observational research, such as single-center, non-randomized, and small-sample-size studies. Therefore, multicenter prospective observational studies are warranted to validate current findings.

In conclusion, compared with systemic treatments such as systemic chemotherapy and radiotherapy, local interventional control treatment for unresectable or recurrent tumors after surgery offers longer OS. On the other hand, local interventional treatment can downstage the tumor and increase the resectability of CCA. Prospective randomized trials are needed to confirm the application range, indications, and target populations who are most likely to benefit from local interventional therapy.

## Author Contributions

J-HG, LC, and HL drafted the manuscript. All authors contributed to the article and approved the submitted version.

## Funding

This study was supported by National Natural Science foundation of China (81520108015, 81827805, and 81971716) and the Jiangsu Provincial Special Program of Social Program of Social Development (BE2016783 and SBK20190350). The funding sources had no role in writing of the report or decision to submit the paper for publication.

## Conflict of Interest

The authors declare that the research was conducted in the absence of any commercial or financial relationships that could be construed as a potential conflict of interest.
